# Effects of different nitrogen applications and straw return depth on straw microbial and carbon and nitrogen cycles in paddy fields in the cool zone

**DOI:** 10.1038/s41598-024-56481-9

**Published:** 2024-03-18

**Authors:** Lin Liu, Ming Cheng, Jingyi Jin, Minjie Fu

**Affiliations:** 1 School of Agriculture, Yanbian University, Yanji, 133002 China; 2 College of Resources and Environment, China Agricultural University, Beijing, 100193 China; 3 Research Center of Chemical Biology, Yanbian University, Yanji, 133002 China

**Keywords:** Nitrogen cycle genes, Carbon cycle genes, Rice ecosystem, Macrogenome, Straw return, Cool zone, Environmental microbiology, Ecology

## Abstract

Straw is an important source of organic fertilizer for soil enrichment, however, the effects of different nitrogen(N) application rates and depths on straw decomposition microorganisms and carbon and nitrogen cycling under full straw return conditions in cool regions of Northeast China are not clear at this stage. In this paper, we applied macro-genome sequencing technology to investigate the effects of different N application rates (110 kg hm^−2^, 120 kg hm^−2^, 130 kg hm^−2^, 140 kg hm^−2^, 150 kg hm^−2^) and depths (0–15 cm, 15–30 cm) on straw decomposing microorganisms and N cycling in paddy fields in the cool zone of Northeast China. The results showed that (1) about 150 functional genes are involved in the carbon cycle process of degradation during the degradation of returned straw, of which the largest number of functional genes are involved in the methane production pathway, about 42, the highest abundance of functional genes involved in the citric acid cycle pathway. There are 22 kinds of functional genes involved in the nitrogen cycle degradation process, among which there are more kinds involved in nitrogen fixation, with 4 kinds. (2) High nitrogen application (150 kg hm^−2^) inhibited the carbon and nitrogen conversion processes, and the abundance of straw-degrading microorganisms and nitrogen-cycling functional genes was relatively high at a nitrogen application rate of 130 kg hm^−2^. (3) Depth-dependent heterogeneity of the microbial community was reduced throughout the vertical space. At 71 days of straw return, the nitrogen cycling function decreased and some carbon functional genes showed an increasing trend with the increase of straw return depth. The nitrogen cycle function decreased with the increase of straw returning depth. The microbial community structure was best and the abundance of functional genes involved in the nitrogen cycling process was higher under the conditions of 0–15 cm of returning depth and 130 kg hm^−2^ of nitrogen application.

## Introduction

Crop straw is an important by-product of the agricultural production process, accounting for about 50% of crop biomass^1^. China is a large agricultural producer and ranks first in the world in terms of annual straw production^2^, accounting for about 30% of the world's total straw^3^. As an important renewable resource, straw contains mineral nutrients and large amounts of organic matter essential for plant growth and is an important source of organic fertilizer for soil enrichment^4^. In agroecosystems, straw return is the most effective way to obtain nitrogen nutrients other than applied nitrogen fertilizer^5^. Therefore, the full utilization of nutrient resources in crop straw is important to reduce chemical fertilizer inputs, improve soil fertility, and maintain sustainable agricultural production in China. Straw decay is a mineralization process and humification process involving microorganisms, which is regulated by soil environment, field management, and tillage depth^6^. As a high carbon and nitrogen material, straw return to the field leads to a large amount of soil inorganic nitrogen sources being sequestered by microorganisms, resulting in microbial competition with crops for nitrogen and affecting soil nitrogen supply, so straw return to the field is often combined with nitrogen fertilizer application^7^. Kanal^8^ et al. found that microbial activity decreased with increasing depth of return to the field.

The carbon (C) and nitrogen (N) cycles are fundamental biogeochemical cycles mediated by microorganisms and have an important regulatory role between soil material dynamics and net gas exchange between the soil and the atmosphere^9^. The balance of the carbon and nitrogen cycles is essential for maintaining soil quality and improving crop growth, and is also relevant to the stability of agroecosystems and improving agricultural productivity^10^. The carbon and nitrogen cycle process of straw degradation after returning to the field is a series of carbon and nitrogen degradation and fixation, denitrification processes, etc. driven by microorganisms. Therefore, understanding the carbon and nitrogen metabolic processes of returned straw is important to determine the bioavailable carbon and nitrogen for sustainable agriculture. Chemical nitrogen fertilization helps agricultural soils to maintain relatively stable levels of soil C fixation and degradation-related gene abundance and inhibits methane oxidation processes in paddy soils^11^. The bioavailability of organic N in soil depends on different transformation reactions in the N cycle, which depends mainly on the composition of soil microbial communities and their functions^12^. Therefore the study of the nitrogen cycle provides not only a theoretical basis for farming systems but also an important reference for biogeochemical cycles mediated by microorganisms. Yao et al.^13^ found that many factors, including land use, soil type, climatic conditions, and nitrogen deposition, synergistically drove the spatial distribution of ammonia-oxidizing microorganisms in a region-wide study in Scotland^14,15^. The results of a study by Jiang et al.^16^ on undisturbed wetland soils indicated that the distribution characteristics of denitrifying microbial communities were significantly influenced by environmental factors.Therefore, even though some studies have been reported on the degradation of returned straw, they still cannot reasonably explain the degradation process of returned straw under flooded conditions in the cool zone.

Previous studies on carbon and nitrogen utilization of straw returned to the field mainly focused on the soil after straw return^17–19^. The effects of straw return measures on carbon and nitrogen functional cycles during straw's own degradation have not been reported. In this study, we investigated the effects of nitrogen application and straw depth on soil microbial and nitrogen cycling in the cooler regions of northeast China and analyzed the gene abundance of key processes of carbon and nitrogen cycling during straw decomposition and straw return to the field using in situ culture of straw in nylon mesh bags combined with macro-genome sequencing. The aim of this study is to analyze the changes in gene abundance of key carbon and nitrogen cycling processes and the similarities and differences in the responses of decomposing microorganisms to field restoration measures and to reveal the distribution characteristics of carbon and nitrogen cycling functional microorganism genes, which is important for straw field restoration in cold and cool regions.

## Materials and methods

### Study site

The experiment was located in 2021 at the experimental base of Yanbian University, which belongs to the single-season rice-winter recreational cropping system. The average annual precipitation in the test area is 549.3 mm, the average annual temperature is 5.6 °C, and the frost-free period is 126 days. Due to the combined influence of oceanic climate and terrestrial climate, weather below 18 °C often occurs for many days in July, and the annual active temperature is about 2650 °C, which belongs to the cool zone in the northeast. The test soil was submerged rice soil. The basic physical and chemical properties of the soil are shown in Table 1.

**Table 1:** Basic physicochemical properties of soil samples.

Soil horizon	pH	EC	Available K	NO_3_^−^-N	NH_4_^+^-N	Available P	Total K	Total N	Total P	Organic matter
		(μs/cm)	(mg/kg)	(mg/kg)	(mg/kg)	(mg/kg)	(g/kg)	(g/kg)	(g/kg)	(g/kg)
0–15 cm	6.12	131.03	485.00	23.46	69.35	33.17	16.71	0.73	1.12	62.91
15–30 cm	6.69	82.27	227.00	22.35	40.22	19.19	19.21	0.49	0.95	59.53

### Experiments

N fertilizer (urea: 46% N) was applied at a rate of 4:4:2 for each treatment: base fertilizer: tiller fertilizer: spike fertilizer. All treatments applied phosphorus (diammonium phosphate: 18% N, 46% P_2_O_5_) and potassium (potassium sulfate: 50% K_2_O) in the same amount and at the same time, with all phosphorus (P_2_O_5_, 70 kg hm^−2^) applied as basal fertilizer and potassium (K_2_O, 80 kg hm^−2^) applied as basal fertilizer: tiller fertilizer: spike fertilizer 5:4:1. The straw was shredded and left in the field in autumn of the previous year, and then rototilled and returned to the field in spring of the following year. Other field management measures were consistent with local conventional management.

The straw decomposition test was carried out using the straw bale in situ stratified culture method. The rice straw after yellow maturation in the previous year was cut into 5 cm length and weighed with a dry weight of 13.75 g of straw in a nylon mesh bag (size: 10 cm width, 30 cm length, 0.1 mm aperture). The straw bags were buried diagonally at ∠45° in the soil layers of 0–15 cm (TU) and 15–30 cm (TD) in the tillage layer and sub-tillage layer, respectively, before planting in spring. In combination with the nitrogen application level, 10 treatments were set up at 110 kg hm^−2^ (TU1, TD1), 120 kg hm^−2^ (TU2, TD2), 130 kg hm^−2^ (TU3, TD3), 140 kg hm^−2^ (TU4, TD4), 150 kg hm^−2^ (TU5, TD5), and the straw samples were removed from each treatment on the 71st day of the experiment (rice tillering stage). The straw samples were transported on dry ice to the laboratory for microbiological analysis.

### Determination of straw nutrient content

The determination of total nitrogen content of straw was carried out by -cooking-AA3 flow analyzer, the determination of total phosphorus content of substrate was carried out by -cooking-vanadium-molybdenum-yellow colorimetric method, and the determination of total potassium content of substrate was carried out by -cooking-flame photometric method. The straw organic matter content was determined by concentrated sulfuric acid-permanganate heating method. Measurement methods were referred to those of Baoshidan.

### Sample DNA extraction

Straw microbiome DNA was extracted by the PowerSoil DNA Isolation Kit (MoBio Laboratories, Inc., CA) and the DNA concentration of the samples was accurately quantified by Qubit assay.

### Library construction and sequencing

The samples were randomly broken into 350 bp fragments by Covaris ultrasonic fragmentation, and the library was prepared by end repair, A-tail addition, sequencing junction addition, purification, PCR amplification, and other steps. After the library was constructed, the library was initially quantified using Qubit2.0 and diluted to 2 ng ul^−1^, and then the insert size of the library was checked using Agilent 2100. Quality After passing the library test, the libraries are pooled according to the effective concentration and the target downstream data volume and then sequenced on the Illumina NovaSeq4000 platform.

### Data processing and analysis

Raw sequencing sequences, i.e. Raw reads, often contain low-quality Reads with adapters, which are filtered to ensure the quality of information analysis. The criteria and principles of the QC section are as follows: (1) filter the reads with sequencing adapters; (2) filter the reads with N (uncertain bases) content greater than 1%; (3) filter the reads with low-quality bases (Q ≤ 20) content greater than 50%; and filter the reads with fragment length less than 150 bp after QC. The high-quality Clean Reads were compared and species annotated using the DIAMOND BLASTX algorithm, and the sequenced samples were assembled using the assembly software MEGAHIT (v1.0.6)^20^, and fragments below 500 bp were filtered out from the assembly results. The sequenced sequences were predicted by ORF (Open Reading Frame) using Prodigal^21^ software, and the non-redundant gene sets were obtained by redundancy removal with 0.95 similarities using CD-HIT^22^ software, and the sequencing data were compared with the constructed non-redundant gene sets using Bowtie^23^ software. The abundance information of individual genes in different samples was calculated and normalized to obtain the gene abundance table. The nr database (RefSeq non-redundant proteins) was compared using Diamond^24^ based on Reads, combined with Megan6^25^ H. analysis for species composition analysis and Reads-based MetaPhlAN comparison analysis, and based on the results of nr annotation, Based on the results of nr annotation, the dominant microorganisms were screened and correlation analysis was performed. Non-redundant gene sequences were compared with the KEGG database (Kyoto Encyclopedia of Genes and Genomes) with the help of Kobas software, and target genes associated with C and N cycles were screened based on the main pathways of C and N cycles that occurred simultaneously in all macroeconomic samples (Table S1). Statistical analysis of the obtained results was performed.

### Statistical analysis

The data were processed using Excel 2019 and plotted using Origin2021, using statistical methods of analysis to plot the species composition Barplot to observe the community structure and its variation at the level of the sample genus^26^.

### Statement

All rice straw samples collected in this study have been licensed. All the rice straw experiments were in compliance with relevant institutional, national, and international guidelines and legislation.

## Results

### Nutrient composition of straw returned to the field in cooler areas

As shown in Table S2, the total potassium content of returned straw was higher at 110–120 kg hm^−2^ of N application (TU1, TD1, TU2, TD2), and increased with increasing depth of returned straw, but the difference was not significant. The organic matter content of the returned straw was generally higher at low N application rates (110–130 kg hm^−2^). The total N content of returned straw was generally higher in the 15–30 cm soil layer compared to 0–15 cm, with the highest N content in the 130 kg hm^−2^ applied N. The total phosphorus content of TU1 was significantly higher than that of TD1, and the total phosphorus content of returned straw was generally higher in the top soil (0–15 cm), but the difference was not significant. The total phosphorus content of straw was higher at 140 kg hm^−2^ N application (TU4, TD4). The differences in organic matter of returned straw at different depths of return were not significant, and the organic matter content of returned straw was lower at high N application rates (140–150 kg hm^−2^).

### Response of straw-decomposing microorganisms to nitrogen application and depth of return to the field in the cool zone

As shown in Fig. S1, Aspergillus, Penicillium, and Talaromyces were the dominant microorganisms at the genus level in the degradation process of returned straw. The relative abundance of Aspergillus decreased with increasing depth. The relative abundance of the three dominant microorganisms was highest at 130 kg hm^−2^ (TU3, TD3) of nitrogen application. As shown in Fig. S2, the relative abundance of Sphaerobolus, Oidiodendron, Bipolaris, Phialocephala, Coniochaeta, Trichoderma, Chaetomium, Penicillium, Penicillium, Pecoramyces, Talaromyces, and Aspergillus correlations decreased with increasing depth of field return and were higher at 130 kg hm-2 (TU3, TD3) of nitrogen application. Pseudogymnoascus, Colletolrichum, Serendpita, Fusarium, Linnermannia, Trichoderma (Mucor), Batrachochytrium, Spizellomyces, Puccinia, Lichthemia, Verticillium, Allomyces, Rhizopus, Rhizoclonia were more correlated with the 15–30 cm treatments (TD1, TD2, TD3, TD4, TD5) and with low N application rates (110–120 kg hm^−2^).

As shown in Fig. S3, the content of total phosphorus in straw had a significant positive correlation with Coniochaeta, Sphaerobolus, Pecoramyces, Trichoderma, Talaromyces, Penicillium, and Aspergillus. Oidiodendron was significantly and negatively correlated with organic matter content in straw. Talaromyces, Penicillium, and Aspergillus were positively correlated with total nitrogen, phosphorus, and potassium content in straw, but negatively correlated with organic matter content.

### Carbon cycle process of degradation of returned straw

**Figure 1:**
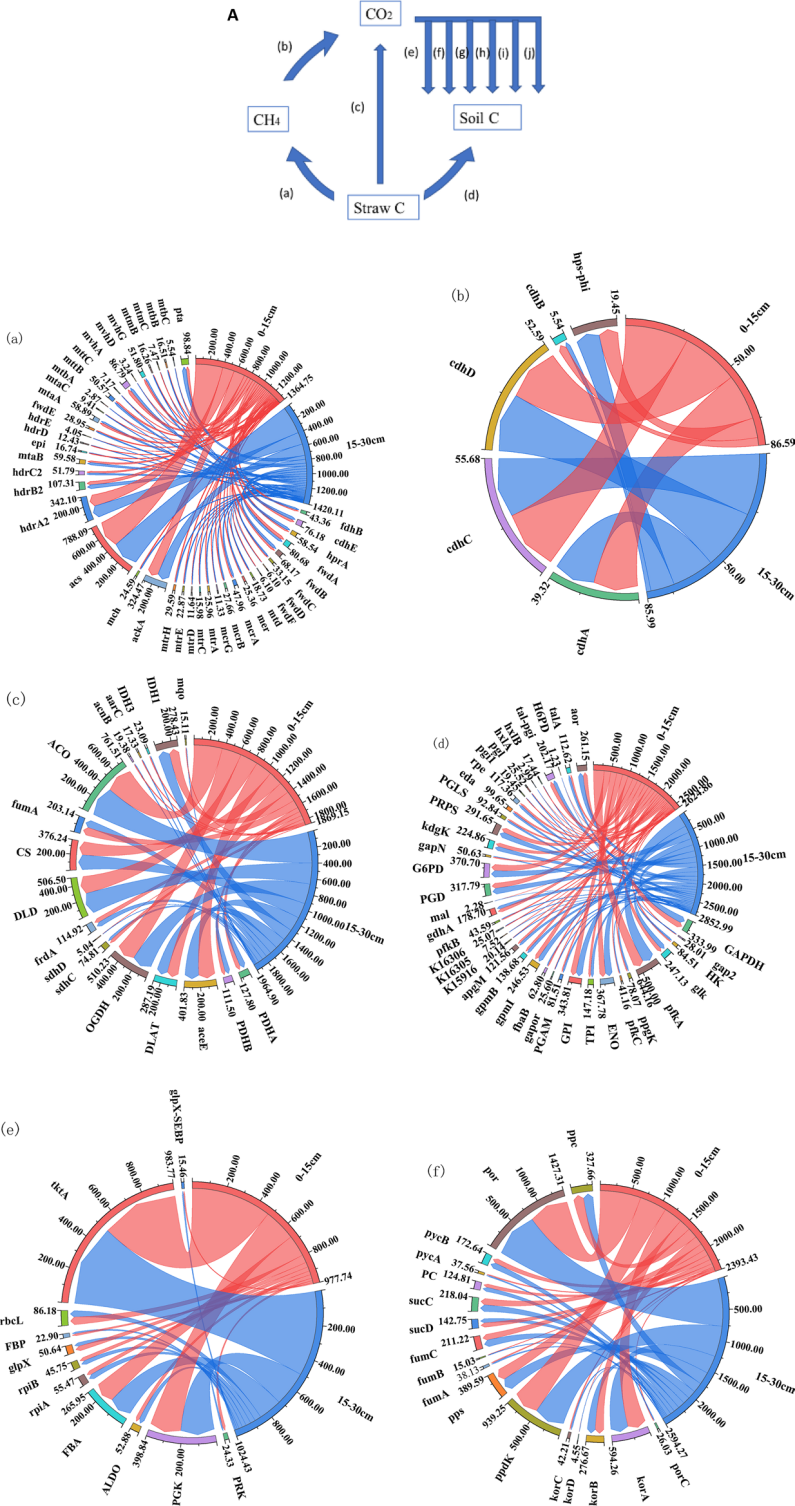

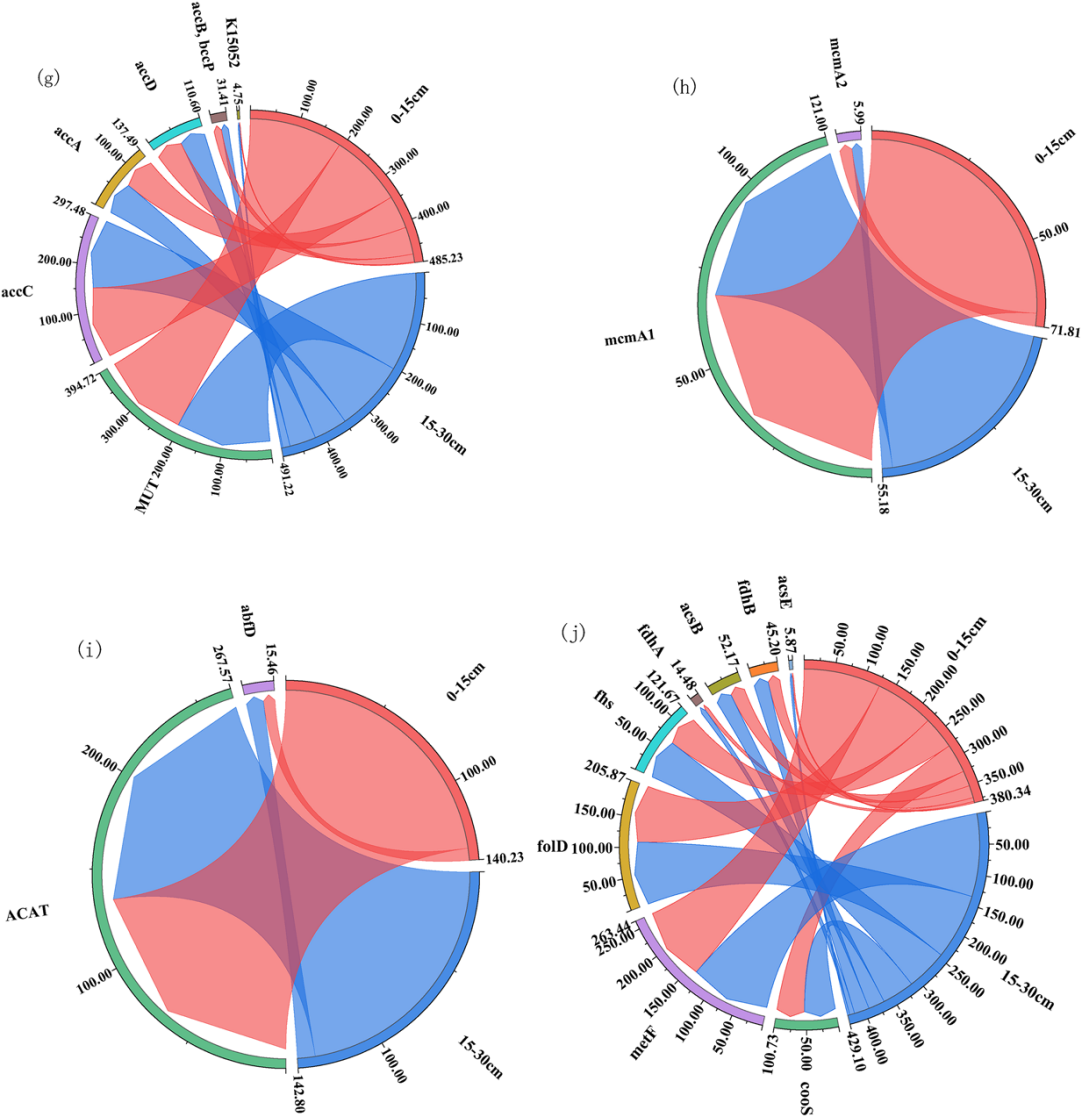
Carbon cycle of degradation of returned straw at different return depths. A indicates the carbon cycle flow diagram, (**a**–**j**) ask the copy number of genes associated with different processes of the carbon cycle in 10^5^/g. The right side of the string diagram shows each treatment, and the copy number of each functional gene is shown on the left side. Clockwise in each functional gene, the copy number of that functional gene decreases sequentially for each treatment.

All genes involved in the C cycle of straw degradation by returning to the field were classified into C degradation, C fixation, and methane metabolism (Fig. 1A). The highest abundance of genes for C utilization-related processes and the largest variety of functional genes were found at different return depths (Fig. 41a,c,d)). The abundance of functional genes associated with the Calvin cycle, reduced citrate cycle processes, and 3-hydroxypropionate double cycle in the carbon fixation system was higher than those associated with the hydroxypropionate-hydroxybutyrate cycle, dicarboxylic acid-hydroxybutyrate cycle, and reduced acetyl coenzyme A pathway (Fig. 1e–j). The highest gene abundance was found for ACSS1-2, ACO, and por during carbon degradation (Fig. 1a,c,d). The highest abundance of ACSS1-2 was found in the methanogenesis process, and the increase of returning depth would promote the production of methane gas during straw degradation (Fig. 1a). 0–15 cm returned straw was more favorable for the oxidation of methane gas, and cdhA, cdhC, and cdhD were the genes in this stage (Fig. 1b). At 71 days of straw return, the abundance of functional genes for carbon utilization was higher in 15–30 cm returned straw compared to 0–15 cm, with a higher copy number of ACO (Fig. 1c). The abundance of carbon fixation genes in the carbon fixation system of straw returned to the field was higher in the straw returned to the field depth of 15–30 cm except for the hydroxypropionic acid-hydroxybutyric acid cycle, E2.2.2.1, por, MUT, E5.4.99.2A, ACAT, and metF for the carbon degradation process of Calvin cycle, reduced citrate cycle process, 3-hydroxypropionic acid double cycle, hydroxypropionic acid-hydroxy butyric acid cycle, dicarboxylic acid-hydroxybutyric acid cycle, and high copy number genes of the reduced acetyl coenzyme A pathway (Fig. 4e–j).

**Figure 2:**
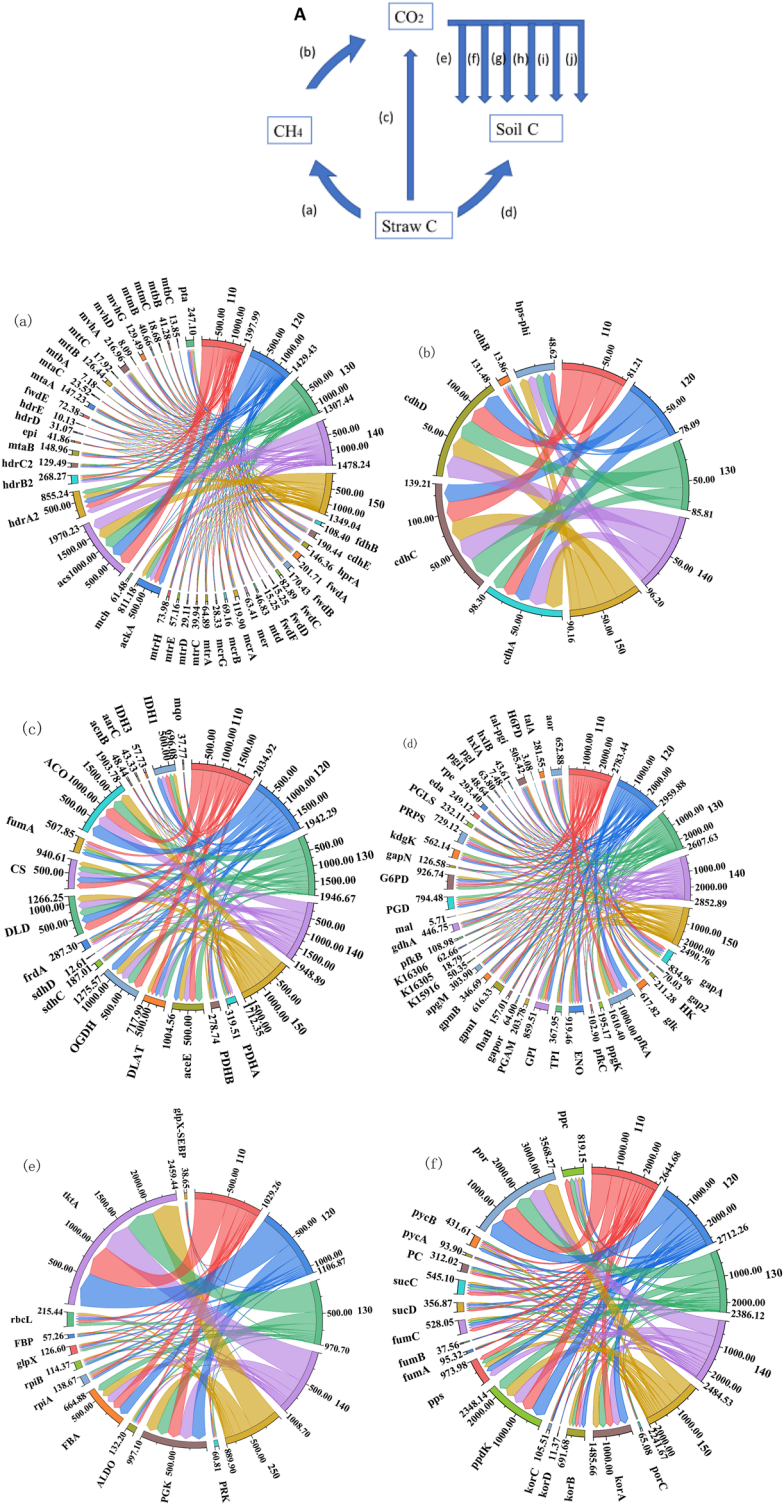

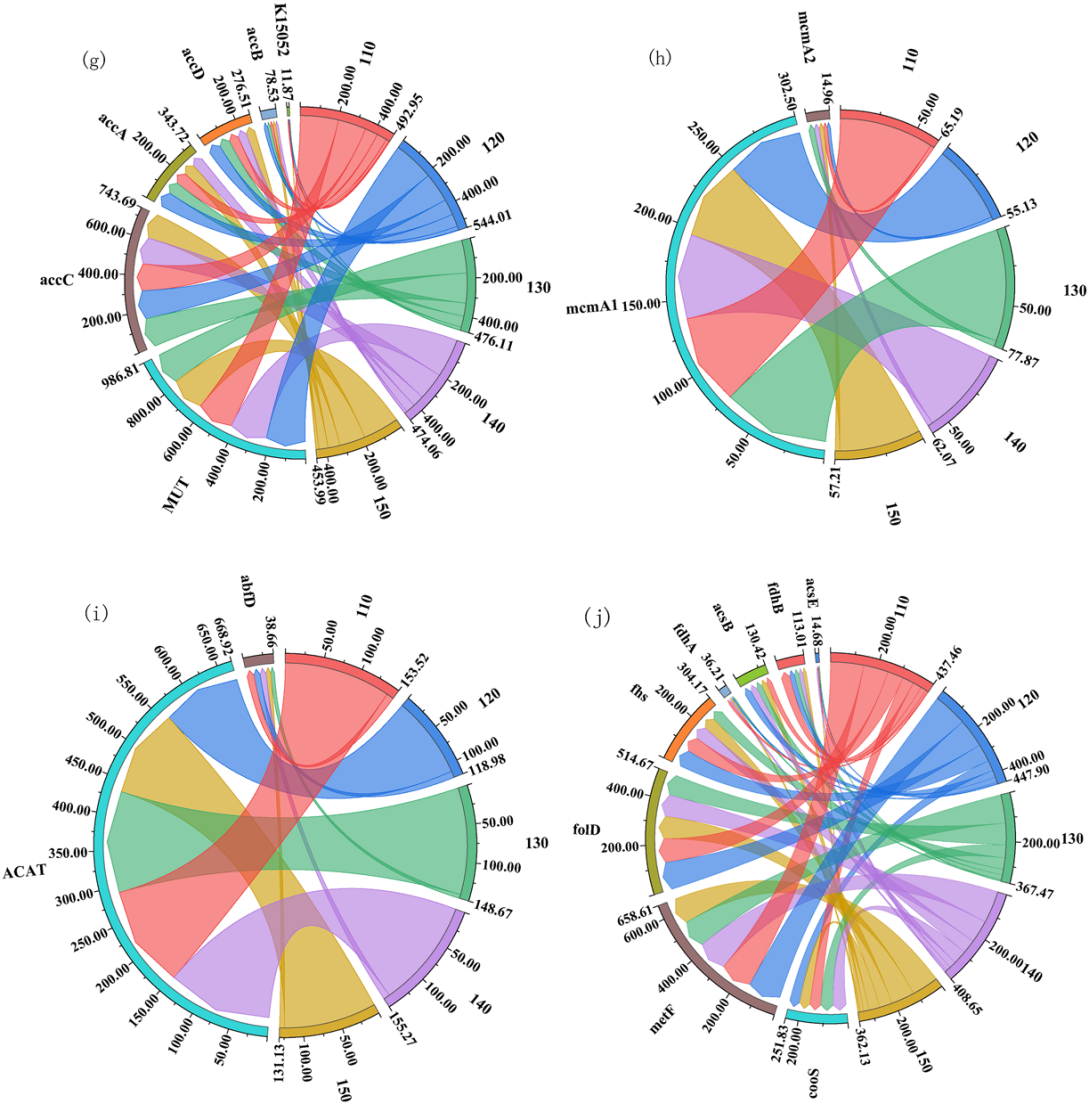
Carbon cycle of degradation of returned straw under different N application. A indicates the carbon cycle flow diagram, (**a**–**j**) ask the copy number of genes associated with different processes of the carbon cycle in 10^5^ g^−1^. The right side of the string diagram shows each treatment, and the copy number of each functional gene is shown on the left side. Clockwise in each functional gene, the copy number of that functional gene decreases sequentially for each treatment.

At low nitrogen application rates (110–130 kg hm^−2^), the number of copies of functional genes involved in methane production during straw degradation decreased with increasing nitrogen application, and about 42 functional genes were involved in this process. In general, the highest copy number of functional genes was found at 140 kg hm^−2^ under different N application rates (Fig. 2a). The highest copy number of functional genes was found at 140 kg hm^−2^ (Fig. 2a). The gene abundance of hps-phi increased with the increase of nitrogen application during methane oxidation, and the highest copy number of cdhC was found at 130 kg hm^−2^ of nitrogen application in this stage (Fig. 2b). The copy number of functional genes of straw degradation carbon utilization pathway was generally lower under high nitrogen application than under low nitrogen application, among which PDHA, PDHB, sdhD, CS, and IDH1 all had the highest copy number at 140 kg hm^−2^ nitrogen application (Fig. 2c). A total of 40 functional genes were involved in the methane oxidation of willow straw, with the highest copy numbers of HK, glk, GPI, gpmI, apgM, por, PRPS, eda, pg1, hxlA, and H6PD at 120 kg hm^−2^ N application, and the highest copy numbers of K16305, K16306, pfkB, GLUD1-2, and G6PD at 140 kg hm^−2^ N application (Fig. 2d). The best performance of the carbon fixation system of the returned straw was achieved by the application of N at 120 kg hm^−2^ and 140 kg hm^−2^ functional genes, with the highest relative abundance of the reduced citrate cycle process functional genes and the most diverse functional genes (Fig. 2e–j).

### Nitrogen cycle process of straw degradation in returned fields

**Figure 3:**
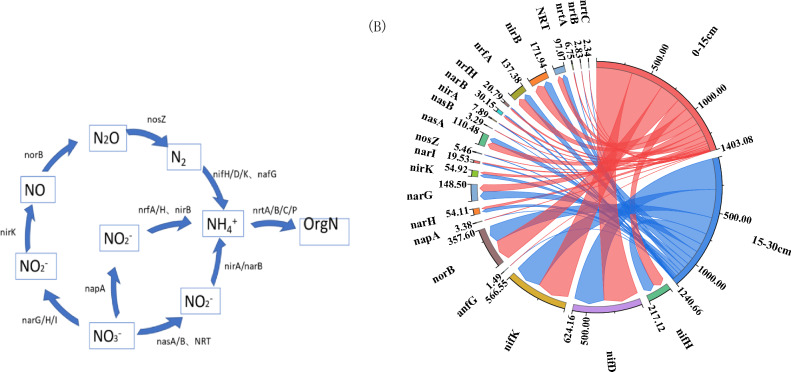
Nitrogen cycle of returned straw degradation under different depths of return. A indicates the process of nitrogen cycle degradation by returned straw, and B indicates the copy number of nitrogen cycle-related genes in 10^5^ g^-1^. The right side of the string diagram shows each treatment, and the copy number of each functional gene is shown on the left side. Clockwise in each functional gene, the copy number of that functional gene decreases for each treatment in order.

As shown in Fig. 3A, nitrification and assimilative nitrate reduction are important processes in the nitrogen cycle of straw decay. The gene copy number of nifD and nifK, which are involved in nitrogen fixation reactions during the nitrogen cycle, was the highest (Fig. 3B), and the gene copy number of anfG was the lowest. norB, nasA, nirB and nrtP, which are the representative functional genes for nitrification, assimilated nitrate reduction, anisotropic nitrate reduction, and nitrate assimilation, respectively, had the highest gene copy number in each process. In addition, the copy number of nitrogen cycle-related genes involved in straw decomposition generally decreased with increasing straw return depth.

**Figure 4:**
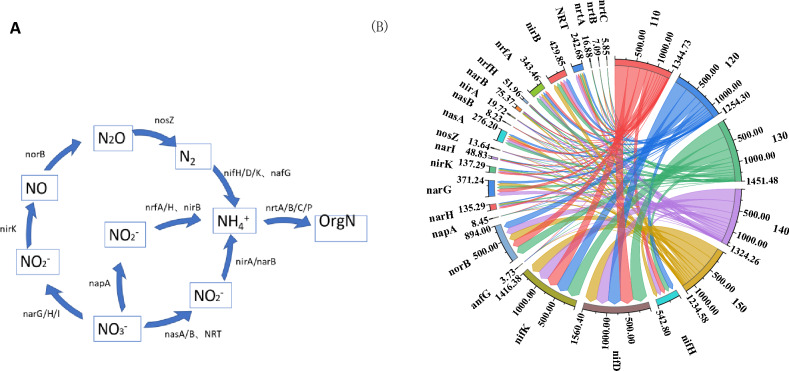
N cycle of degradation of returned straw under different N application rates to the field. A indicates the process of nitrogen cycle degradation by returned straw, and B indicates the copy number of nitrogen cycle-related genes in 10^5^ g^−1^. The right side of the string diagram shows each treatment, and the copy number of each functional gene is shown on the left side. Clockwise in each functional gene, the copy number of that functional gene decreases for each treatment in order.

As shown in Fig. 4, the copy number of genes related to the nitrogen fixation process, nitrification, anabolic nitrate reduction, anisotropic nitrate reduction, and nitrate assimilation were all highest at 130 kg hm^−2^ and second highest at 140 kg hm^−2^. The copy number of each gene (nifH, nifD, nifK, anfG) related to the nitrogen fixation process was the lowest at 150 kg hm^−2^ of nitrogen application.

Figure S4 shows the correlation between the top 30 dominant microorganisms and the carbon and nitrogen cycle processes during straw degradation. Lipomyces, Lichtheimia, Puccinia, Mucor, Verticillium, and Mucor were significantly and positively correlated with the glycolytic process. Puccinia, Mucor, Verticillium, and Plectosphaerellaceae were significantly and positively associated with the pentose phosphate pathway. The tricarboxylic acid cycle was significantly positively correlated with the relative abundance of Rhizoctonia and negatively correlated with Trichoderma. Coniochaeta, and Trichoderma had a significant negative correlation with methane production, and the increase of their relative abundance was beneficial to reduce methane gas emission, and Rhizoctonia had a significant positive correlation with the metabolic process of methane production. Linnermannia, Exophiala, Puccinia, and Mucorall showed significant negative correlations with the oxidation of methane, i.e., CO_2_ production. The increase in the relative abundance of Aureobasidiun, Puccinia, and Verticillium in the small incomplete spherical shell family (Plectosphaerellaceae) during carbon fixation facilitated the Calvin cycle process. The tricarboxylic acid cycle was significantly positively correlated with Lichtheimia, Puccinia, and Plectosphaerellaceaeand negatively correlated with Pecoramyces. The 3-hydroxypyruvate cycle was significantly correlated with Aureobasidiun, rusts (Puccinia), and Pecoramyces were significantly positively correlated. The hydroxypropionic acid-hydroxybutyric acid cycle was significantly and negatively correlated with Coniochaeta, Pecoramyces, Chaetomium, Plectosphaerellaceae, Mollisiaceae, Trichoderma, Talaromyces, Talaromyces, Penicillium, and Aspergillus all had significant positive correlations. The reduced acetyl coenzyme A pathway was significantly and positively correlated with Lichtheimia, Puccinia. It was significantly negatively correlated with Plectosphaerellaceae. The nitrogen fixation process in the nitrogen cycle of straw degradation was significantly positively correlated with the abundance of Aureobasidiun, Penicillium, and Gonapodya, and negatively correlated with Gonapodya. The denitrification process was significantly positively correlated with Aureobasidiun and negatively correlated with Gonapodya. Coniochaeta, Sphaerobolus, Chaetomium, Pecoramyces, Millisiaceae, Trichoderma, Pseudogymnoascus, Aspergillus were significantly positively correlated with assimilated nitrate reduction and dissimilated nitrate reduction, while Lichtheimia, Puccinia, and Plectosphaerellaceae were negatively correlated with assimilated nitrate reduction and dissimilated nitrate reduction. The nitrate assimilation process was only significantly positively correlated with Coniochaeta, Sphaerobolus, Chaetomium, Penicillium, with the strongest correlation with Chaetomium.

As shown in Table S3, straw depth had no significant effect on the carbon cycle processes of straw degradation for methane oxidation, but had significant effects on methanogenesis, 3-hydroxypyruvate cycle, and dicarboxylic acid-hydroxybutyrate cycle, while the rest of the carbon cycle processes had highly significant effects. Nitrogen cycling processes responded positively to the depth of return, except for N fixation, which was highly significant. The amount of nitrogen applied and its coupling with the depth of return had a significant effect on the carbon and nitrogen cycles of straw degradation.

## Discussion

### Straw-degrading microorganisms in black soil cool areas

The straw decay process is a microbially dominated biochemical process^27,28^. Microorganisms change their community structure with the chemical composition of the residues at different periods of straw decomposition^29–33^. Microbial community diversity determines ecosystem functions and services, and soils in intensive agroecosystems are influenced by microbial diversity^34,35^. Bacteria are the main microorganisms that dominate the degradation of straw during its degradation (Figs. S1, S2). It has been shown that bacteria favor the utilization of easily decomposed plant residues, and fungi grow slower than bacteria and favor the decomposition of difficult compounds^29,36,37^. Saprophytic fungi are an important microbial group that can form branched mycelial communities between the soil-apoptotic interface, linking lignin and cellulose while releasing extracellular enzymes^38^. In this study, it was found that straw-decomposing microorganisms were more sensitive to different depths of return compared to different N applications (Fig. S2), presumably due to the influence of microbial communities under human management, which reduced the depth-dependent heterogeneity of microbial communities across the vertical space^39^. The microbial community did not show regular changes among treatments at different N application rates, but overall the microbial abundance of dominant genera was higher at 130 kg hm^−2^ and 140 kg hm^−2^ N application rates, and there were more correlated microbial species (Figs. S1, S2). High N application (150 kg hm^−2^) inhibited microbial correlations to some extent (Fig. S2), which is similar to previous studies^40^. Many studies have shown that degradation conditions are environmental factors that drive microbial community structure, such as pH, total nitrogen, total carbon, total phosphorus, and organic matter^41,42^. In this experiment, the total nitrogen, total phosphorus, and total potassium of straw were positively correlated with the degradation of dominant microorganisms (Fig. S3).

### Abundance of functional genes of the carbon cycle in straw returned to the field in the cool zone

The addition of N application can induce changes in soil N status, which can accelerate or inhibit the C turnover process of straw degradation (Table S3)^43,44^. In this study, it was found that the C cycle-related functional genes generally increased with the increase of the depth of the returned straw, which was different from the conclusions of previous studies, and the analysis was due to the fact that the straw degradation process would preferentially decompose soluble substances that are easy to decompose, and gradually decompose cellulose, hemicellulose, and phenolic compounds that are difficult to decompose after the consumption of soluble substances is completed In this period, the soluble substances in the 15–30 cm soil layer that had not yet been completely decomposed by straw were consumed, and the straw in the 0–15 cm soil layer had already entered the next stage of decomposition. About 42 functional genes were involved in methanogenesis in this study, which accounted for a large proportion of functional genes in this carbon cycle, which is similar to the results of previous studies, further indicating that straw decomposition promotes methanogenesis^45,46^. At the same straw return depth, some of the functional genes involved in methanogenesis tended to increase with increasing nitrogen application, and the analysis suggested that the reason for this phenomenon was related to the influence of the returning soil and the climatic conditions of the cool zone on the related microbial metabolism^47^. The present study found that high nitrogen application increased the copy number of methane oxidation genes to some extent, a finding that differs from previous studies, which showed^11^ that chemical nitrogen fertilizer application inhibited the expression of methane oxidation functional genes, presumably due to the fact that high nitrogen application promotes the degradation of straw making different reaction substrates. In this study, we found that even though the preferred more relevant microbial communities differed among treatments at different straw return depths and different nitrogen applications, the functions of each microorganism were similar.

### Abundance of functional genes in the nitrogen cycle of straw returned to the field in the cool zone

The main processes involved in the nitrogen cycle by microorganisms include nitrogen fixation, nitrate assimilation reduction, nitrate allotropic reduction, nitrification and denitrification processes, and biosynthesis and degradation of organic nitrogen^48,49^. The nitrogen cycling processes of returned straw of interest in this study mainly include the nitrogen fixation process, nitrification, assimilative nitrate reduction, allotropic nitrate reduction, and nitrate assimilation, and its functional genes also include regulatory-related functional genes associated with genes encoding related functional proteins. It was found that nitrogen application significantly affected the nitrogen cycling process, and the intensity of nitrogen cycling was higher for each functional gene at 130 kg hm^−2^ and 140 kg hm^−2^ of nitrogen application, and the copy number of related functional genes was reduced and functional genes were repressed at higher nitrogen application (150 kg hm^−2^) (Fig. 4, Table S3). The enzyme inhibition hypothesis suggests that this situation is due to the addition of exogenous nitrogen fertilizer inhibiting the activity of enzymes that break down recalcitrant carbon, therefore reducing the overall metabolic activity of microorganisms, which further inhibits the functional metabolism of N cycle-related pathways under the mechanism of carbon and nitrogen cycle interactions^50^. At low N application rates (110 kg hm^−2^, 120 kg hm^−2^), the N cycle-related genes of straw decomposition decreased with increasing N application, and according to the "microbial N mining theory", microorganisms preferentially utilize mineral N when appropriate N is added and have little incentive to decompose organic matter to obtain N^51^. The N cycle is a superbly broad process mediated by many different microorganisms; therefore, any change in community structure may be associated with changes in N cycling potential^52,53^. In this study, vertical changes in the dominant microbial community may have driven different responses in N cycling potential, with the N cycling function of the decomposition process decreasing with deeper straw return (Fig. 3).

## Conclusions

Based on the study of N application and return depth on straw-decomposing microorganisms and carbon and nitrogen cycling in the cool zone, it was found that both N application and return depth had highly significant effects on carbon and nitrogen cycling processes under the full amount of straw returned to the field. Compared with the N application, the straw-decomposing microorganisms were more sensitive to the depth of return, and the highly correlated microorganisms at different depths of return showed significant partitioning. The highest number of highly correlated microorganisms for straw degradation was observed at 130 kg hm^−2^ N application. ACSS1-2, ACO, and por were the main functional genes for straw decomposition in the returned straw. norB, nasA, nirB, and nrtP were the main functional genes for nitrification, assimilated nitrate reduction, allotropic nitrate reduction, and nitrate assimilation in the returned straw decomposition nitrogen cycle, respectively. With the increase of straw return depth, the nitrogen cycle function of straw decay decreased and the carbon function genes showed an increasing trend at 71 days of return. The carbon and nitrogen cycling capacity were strongest at 130 kg hm^−2^ and 140 kg hm^−2^ of nitrogen application, and high nitrogen application would inhibit the nitrogen cycling capacity to some extent. Therefore, the return of rice straw to the soil in the cool zone is beneficial to the optimization of the microbial community structure and the conversion of organic nitrogen from straw at 0–15 cm and 130 kg hm^−2^ of nitrogen application.

### Supplementary Information

Supplementary Information.

## Data Availability

The raw genomic reads generated in this study have been deposited in the NCBI Sequence Read Archive (Biological Project Number PRJNA1082613 https://www.ncbi.nlm.nih.gov/sra/PRJNA1082613). The datasets generated during and/or analyzed during the current study are available from the corresponding author on reasonable request.
