# Supplementary material for: Effects of different nitrogen applications and straw return depth on straw microbial and carbon and nitrogen cycles in paddy fields in the cool zone
Source: Sci Rep. 2024 Mar 18;14:6424. doi: 10.1038/s41598-024-56481-9 (PMC10944828; doi:10.1038/s41598-024-56481-9)
Supplement: Supplementary file 1 — Supplementary Information. [file 41598_2024_56481_MOESM1_ESM.docx]

**Supplementary Information**

**Effects of straw return coupled with nitrogen application on soil microbial functions of rice fields in a cool zone**

Liu Lin^a^, Cheng Ming^a^, Jingyi Jin ^b^, Fu Minjie ^a,*^

^a^College of Agriculture, Yanbian University, Yanji 133002, China

^b^Research Center of Chemical Biology, Yanbian University, Yanji 133002

Number of Figures: 4 (Pages 2−5)

Number of Tables: 3 (Pages 5−13)

* Corresponding author

E-mail address: [fuminjie@163.com](mailto:fuminjie@163.com) ( M.J.Fu).

**Figure S1.** Microbial abundance map of straw degrading genus levels.


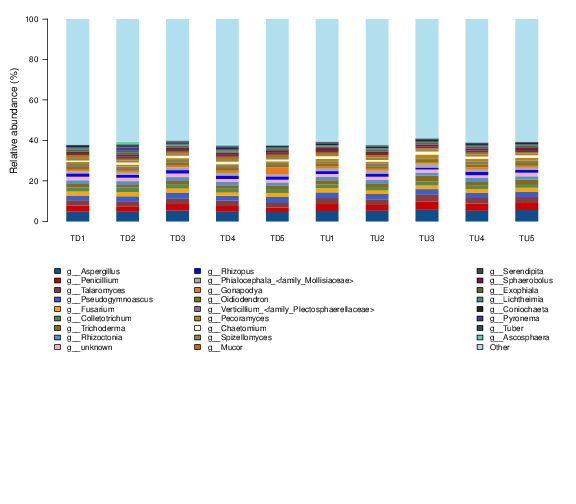


**Figure S2.** Straw degradation microbial correlation heat map. A indicates the return depth of 0-15cm,B indicates the return depth of 15-30cm.


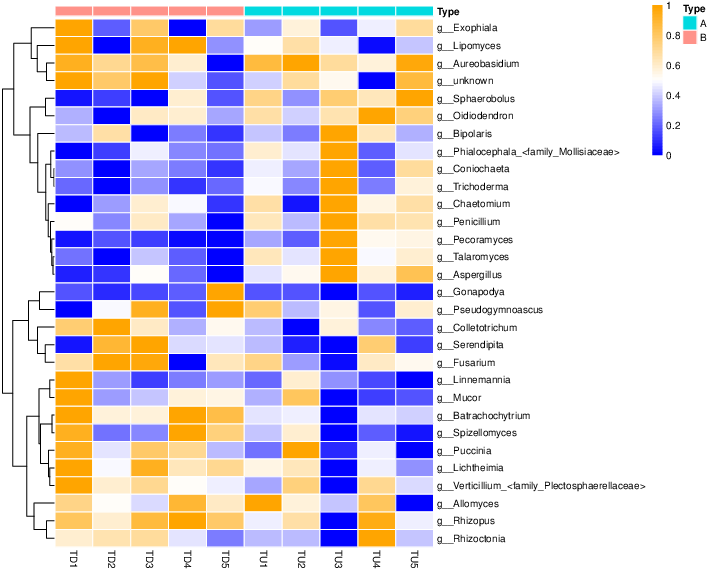


**Figure S3.** Heat map of correlation between straw-degrading dominant microorganisms and straw composition. Red indicates positive correlation, blue indicates negative correlation, the size of the circle indicates the magnitude of the correlation, and * indicates the significance of P < 0.05.

**Figure S4.** Heat map of the correlation between carbon and nitrogen cycles of returned straw and degrading microorganisms. Red indicates positive correlation, blue indicates negative correlation, the size of the circle indicates the magnitude of the correlation, and * indicates the significance of P < 0.05.

**Table S1.** Functional gene information of microorganisms involved in carbon and nitrogen cycling processes was identified in this study.

| **KEGG orthology number** | **Gene name** | **Encoded protein[EC]** |
| --- | --- | --- |
| **Carbon degradation** |  |  |
| **Glycolysis** |  |  |
| K00134 | GAPDH, gapA | glyceraldehyde 3-phosphate dehydrogenase (phosphorylating) [EC:1.2.1.12] |
| K00150 | gap2 | glyceraldehyde-3-phosphate dehydrogenase (NAD(P)) [EC:1.2.1.59] |
| K00844 | HK | hexokinase [EC:2.7.1.1] |
| K00845 | glk | glucokinase [EC:2.7.1.2] |
| K00850 | pfkA, PFK | 6-phosphofructokinase 1 [EC:2.7.1.11] |
| K00873 | PK, pyk | pyruvate kinase [EC:2.7.1.40] |
| K00886 | ppgK | polyphosphate glucokinase [EC:2.7.1.63] |
| K00918 | pfkC | ADP-dependent phosphofructokinase/glucokinase [EC:2.7.1.146 2.7.1.147] |
| K01689 | ENO, eno | enolase [EC:4.2.1.11] |
| K01803 | TPI, tpiA | triosephosphate isomerase (TIM) [EC:5.3.1.1] |
| K01810 | GPI, pgi | glucose-6-phosphate isomerase [EC:5.3.1.9] |
| K01834 | PGAM, gpmA | 2,3-bisphosphoglycerate-dependent phosphoglycerate mutase [EC:5.4.2.11] |
| K11389 | gapor | glyceraldehyde-3-phosphate dehydrogenase (ferredoxin) [EC:1.2.7.6] |
| K11645 | fbaB | fructose-bisphosphate aldolase, class I [EC:4.1.2.13] |
| K15633 | gpmI | 2,3-bisphosphoglycerate-independent phosphoglycerate mutase [EC:5.4.2.12] |
| K15634 | gpmB | 2,3-bisphosphoglycerate-dependent phosphoglycerate mutase [EC:5.4.2.11] |
| K15635 | apgM | 2,3-bisphosphoglycerate-independent phosphoglycerate mutase [EC:5.4.2.12] |
| K15916 | K15916 | glucose/mannose-6-phosphate isomerase [EC:5.3.1.9 5.3.1.8] |
| K16305 | K16305 | fructose-bisphosphate aldolase / 6-deoxy-5-ketofructose 1-phosphate synthase [EC:4.1.2.13 2.2.1.11] |
| K16306 | K16306 | fructose-bisphosphate aldolase / 2-amino-3,7-dideoxy-D-threo-hept-6-ulosonate synthase [EC:4.1.2.13 2.2.1.10] |
| K16370 | pfkB | 6-phosphofructokinase 2 [EC:2.7.1.11] |
| K00161 | PDHA, pdhA | pyruvate dehydrogenase E1 component alpha subunit [EC:1.2.4.1] |
| K00162 | PDHB, pdhB | pyruvate dehydrogenase E1 component beta subunit [EC:1.2.4.1] |
| K00163 | aceE | pyruvate dehydrogenase E1 component [EC:1.2.4.1] |
| K00627 | DLAT, aceF, pdhC | pyruvate dehydrogenase E2 component (dihydrolipoamide acetyltransferase) [EC:2.3.1.12] |
| **Pentose phosphate pathway** | |  |
| K00261 | GLUD1_2, gdhA | glutamate dehydrogenase (NAD(P)+) [EC:1.4.1.3] |
| K03737 | por, nifJ | pyruvate-ferredoxin/flavodoxin oxidoreductase [EC:1.2.7.1 1.2.7.-] |
| K04835 | mal | methylaspartate ammonia-lyase [EC:4.3.1.2] |
| K00164 | OGDH, sucA | 2-oxoglutarate dehydrogenase E1 component [EC:1.2.4.2] |
| K00241 | sdhC, frdC | succinate dehydrogenase cytochrome b subunit |
| K00242 | sdhD, frdD | succinate dehydrogenase membrane anchor subunit |
| K00244 | frdA | succinate dehydrogenase flavoprotein subunit [EC:1.3.5.1] |
| K00382 | DLD, lpd, pdhD | dihydrolipoamide dehydrogenase [EC:1.8.1.4] |
| K01647 | CS, gltA | citrate synthase [EC:2.3.3.1] |
| K01676 | E4.2.1.2A, fumA, fumB | fumarate hydratase, class I [EC:4.2.1.2] |
| K01681 | ACO, acnA | aconitate hydratase [EC:4.2.1.3] |
| K01682 | acnB | aconitate hydratase 2 / 2-methylisocitrate dehydratase [EC:4.2.1.3 4.2.1.99] |
| K18118 | aarC, cat1 | succinyl-CoA:acetate CoA-transferase [EC:2.8.3.18] |
| K00030 | IDH3 | isocitrate dehydrogenase (NAD+) [EC:1.1.1.41] |
| K00031 | IDH1, IDH2, icd | isocitrate dehydrogenase [EC:1.1.1.42] |
| K00116 | mqo | malate dehydrogenase (quinone) [EC:1.1.5.4] |
| **Citric acid cycle** |  |  |
| K00033 | PGD, gnd, gntZ | 6-phosphogluconate dehydrogenase [EC:1.1.1.44 1.1.1.343] |
| K00036 | G6PD, zwf | glucose-6-phosphate 1-dehydrogenase [EC:1.1.1.49 1.1.1.363] |
| K00131 | gapN | glyceraldehyde-3-phosphate dehydrogenase (NADP+) [EC:1.2.1.9] |
| K00874 | kdgK | 2-dehydro-3-deoxygluconokinase [EC:2.7.1.45] |
| [K00948](http://webvpn.ybu.edu.cn/) | PRPS, prsA | ribose-phosphate pyrophosphokinase [EC:2.7.6.1] |
| K01057 | PGLS, pgl, devB | 6-phosphogluconolactonase [EC:3.1.1.31] |
| K01625 | eda | 2-dehydro-3-deoxyphosphogluconate aldolase / (4S)-4-hydroxy-2-oxoglutarate aldolase [EC:4.1.2.14 4.1.3.42] |
| K01783 | rpe, RPE | ribulose-phosphate 3-epimerase [EC:5.1.3.1] |
| K06859 | pgi1 | glucose-6-phosphate isomerase, archaeal [EC:5.3.1.9] |
| K07404 | pgl | 6-phosphogluconolactonase [EC:3.1.1.31] |
| K08093 | hxlA | 3-hexulose-6-phosphate synthase [EC:4.1.2.43] |
| K08094 | hxlB | 6-phospho-3-hexuloisomerase [EC:5.3.1.27] |
| K13810 | tal-pgi | transaldolase / glucose-6-phosphate isomerase [EC:2.2.1.2 5.3.1.9] |
| K13937 | H6PD | hexose-6-phosphate dehydrogenase [EC:1.1.1.47 3.1.1.31] |
| K00616 | E2.2.1.2, talA, talB | transaldolase [EC:2.2.1.2] |
| K03738 | aor | aldehyde:ferredoxin oxidoreductase [EC:1.2.7.5] |
| **Methane metabolism** |  |  |
| **Methanogenesis** |  |  |
| K00125 | fdhB | formate dehydrogenase (coenzyme F420) beta subunit [EC:1.17.98.3 1.8.98.6] |
| K00197 | cdhE, acsC | acetyl-CoA decarbonylase/synthase, CODH/ACS complex subunit gamma [EC:2.1.1.245] |
| K00018 | hprA | glycerate dehydrogenase [EC:1.1.1.29] |
| K00200 | fwdA, fmdA | formylmethanofuran dehydrogenase subunit A [EC:1.2.7.12] |
| K00201 | fwdB, fmdB | formylmethanofuran dehydrogenase subunit B [EC:1.2.7.12] |
| K00202 | fwdC, fmdC | formylmethanofuran dehydrogenase subunit C [EC:1.2.7.12] |
| K00203 | fwdD, fmdD | formylmethanofuran dehydrogenase subunit D [EC:1.2.7.12] |
| K00205 | fwdF, fmdF | 4Fe-4S ferredoxin |
| K00319 | mtd | methylenetetrahydromethanopterin dehydrogenase [EC:1.5.98.1] |
| K00320 | mer | 5,10-methylenetetrahydromethanopterin reductase [EC:1.5.98.2] |
| K00399 | mcrA | methyl-coenzyme M reductase alpha subunit [EC:2.8.4.1] |
| K00401 | mcrB | methyl-coenzyme M reductase beta subunit [EC:2.8.4.1] |
| K00402 | mcrG | methyl-coenzyme M reductase gamma subunit [EC:2.8.4.1] |
| K00577 | mtrA | tetrahydromethanopterin S-methyltransferase subunit A [EC:2.1.1.86] |
| K00579 | mtrC | tetrahydromethanopterin S-methyltransferase subunit C [EC:2.1.1.86] |
| K00580 | mtrD | tetrahydromethanopterin S-methyltransferase subunit D [EC:2.1.1.86] |
| K00581 | mtrE | tetrahydromethanopterin S-methyltransferase subunit E [EC:2.1.1.86] |
| K00584 | mtrH | tetrahydromethanopterin S-methyltransferase subunit H [EC:2.1.1.86] |
| K00925 | ackA | acetate kinase [EC:2.7.2.1] |
| K01499 | mch | methenyltetrahydromethanopterin cyclohydrolase [EC:3.5.4.27] |
| K01895 | ACSS1_2, acs | acetyl-CoA synthetase [EC:6.2.1.1] |
| K03388 | hdrA2 | heterodisulfide reductase subunit A2 [EC:1.8.7.3 1.8.98.4 1.8.98.5 1.8.98.6] |
| K03389 | hdrB2 | heterodisulfide reductase subunit B2 [EC:1.8.7.3 1.8.98.4 1.8.98.5 1.8.98.6] |
| K03390 | hdrC2 | heterodisulfide reductase subunit C2 [EC:1.8.7.3 1.8.98.4 1.8.98.5 1.8.98.6] |
| K04480 | mtaB | methanol---5-hydroxybenzimidazolylcobamide Co-methyltransferase [EC:2.1.1.90] |
| K05606 | MCEE, epi | methylmalonyl-CoA/ethylmalonyl-CoA epimerase [EC:5.1.99.1] |
| K08264 | hdrD | heterodisulfide reductase subunit D [EC:1.8.98.1] |
| K08265 | hdrE | heterodisulfide reductase subunit E [EC:1.8.98.1] |
| K11261 | fwdE, fmdE | formylmethanofuran dehydrogenase subunit E [EC:1.2.7.12] |
| K14080 | mtaA | [methyl-Co(III) methanol/glycine betaine-specific corrinoid protein]: coenzyme M methyltransferase [EC:2.1.1.246 2.1.1.377] |
| K14081 | mtaC | methanol corrinoid protein |
| K14082 | mtbA | [methyl-Co(III) methylamine-specific corrinoid protein]:coenzyme M methyltransferase [EC:2.1.1.247] |
| K14083 | mttB | trimethylamine---corrinoid protein Co-methyltransferase [EC:2.1.1.250] |
| K14084 | mttC | trimethylamine corrinoid protein |
| K14126 | mvhA, vhuA, vhcA | F420-non-reducing hydrogenase large subunit [EC:1.12.99.- 1.8.98.5] |
| K14127 | mvhD, vhuD, vhcD | F420-non-reducing hydrogenase iron-sulfur subunit [EC:1.12.99.- 1.8.98.5 1.8.98.6] |
| K14128 | mvhG, vhuG, vhcG | F420-non-reducing hydrogenase small subunit [EC:1.12.99.- 1.8.98.5] |
| K16176 | mtmB | methylamine---corrinoid protein Co-methyltransferase [EC:2.1.1.248] |
| K16177 | mtmC | monomethylamine corrinoid protein |
| K16178 | mtbB | dimethylamine---corrinoid protein Co-methyltransferase [EC:2.1.1.249] |
| K16179 | mtbC | dimethylamine corrinoid protein |
| K00625 | pta | phosphate acetyltransferase [EC:2.3.1.8] |
| **Methane oxidation** |  |  |
| K00192 | cdhA | anaerobic carbon-monoxide dehydrogenase, CODH/ACS complex subunit alpha [EC:1.2.7.4] |
| K00193 | cdhC | acetyl-CoA decarbonylase/synthase, CODH/ACS complex subunit beta [EC:2.3.1.169] |
| K00194 | cdhD, acsD | acetyl-CoA decarbonylase/synthase, CODH/ACS complex subunit delta [EC:2.1.1.245] |
| K00195 | cdhB | anaerobic carbon-monoxide dehydrogenase, CODH/ACS complex subunit epsilon |
| K13831 | hps-phi | 3-hexulose-6-phosphate synthase / 6-phospho-3-hexuloisomerase [EC:4.1.2.43 5.3.1.27] |
| **Carbon fixation** |  |  |
| **Calvin Cycle** |  |  |
| K00855 | PRK, prkB | phosphoribulokinase [EC:2.7.1.19] |
| K00927 | PGK, pgk | phosphoglycerate kinase [EC:2.7.2.3] |
| K01623 | ALDO | fructose-bisphosphate aldolase, class I [EC:4.1.2.13] |
| K01624 | FBA, fbaA | fructose-bisphosphate aldolase, class II [EC:4.1.2.13] |
| K01807 | rpiA | ribose 5-phosphate isomerase A [EC:5.3.1.6] |
| K01808 | rpiB | ribose 5-phosphate isomerase B [EC:5.3.1.6] |
| K02446 | glpX | fructose-1,6-bisphosphatase II [EC:3.1.3.11] |
| K03841 | FBP, fbp | fructose-1,6-bisphosphatase I [EC:3.1.3.11] |
| K01601 | rbcL, cbbL | ribulose-bisphosphate carboxylase large chain [EC:4.1.1.39] |
| K00615 | E2.2.1.1, tktA, tktB | transketolase [EC:2.2.1.1] |
| K11532 | glpX-SEBP | fructose-1,6-bisphosphatase II / sedoheptulose-1,7-bisphosphatase [EC:3.1.3.11 3.1.3.37] |
| **Reduced citrate cycle** |  |  |
| K00172 | porC, porG | pyruvate ferredoxin oxidoreductase gamma subunit [EC:1.2.7.1] |
| K00174 | korA, oorA, oforA | 2-oxoglutarate/2-oxoacid ferredoxin oxidoreductase subunit alpha [EC:1.2.7.3 1.2.7.11] |
| K00175 | korB, oorB, oforB | 2-oxoglutarate/2-oxoacid ferredoxin oxidoreductase subunit beta [EC:1.2.7.3 1.2.7.11] |
| K00176 | korD, oorD | 2-oxoglutarate ferredoxin oxidoreductase subunit delta [EC:1.2.7.3] |
| K00177 | korC, oorC | 2-oxoglutarate ferredoxin oxidoreductase subunit gamma [EC:1.2.7.3] |
| K01006 | ppdK | pyruvate, orthophosphate dikinase [EC:2.7.9.1] |
| K01007 | pps, ppsA | pyruvate, water dikinase [EC:2.7.9.2] |
| K01677 | E4.2.1.2AA, fumA | fumarate hydratase subunit alpha [EC:4.2.1.2] |
| K01678 | E4.2.1.2AB, fumB | fumarate hydratase subunit beta [EC:4.2.1.2] |
| K01679 | E4.2.1.2B, fumC, FH | fumarate hydratase, class II [EC:4.2.1.2] |
| K01902 | sucD | succinyl-CoA synthetase alpha subunit [EC:6.2.1.5] |
| K01903 | sucC | succinyl-CoA synthetase beta subunit [EC:6.2.1.5] |
| K01958 | PC, pyc | pyruvate carboxylase [EC:6.4.1.1] |
| K01959 | pycA | pyruvate carboxylase subunit A [EC:6.4.1.1] |
| K01960 | pycB | pyruvate carboxylase subunit B [EC:6.4.1.1] |
| K03737 | por, nifJ | pyruvate-ferredoxin/flavodoxin oxidoreductase [EC:1.2.7.1 1.2.7.-] |
| K01595 | ppc | phosphoenolpyruvate carboxylase [EC:4.1.1.31] |
| **3-Hydroxypropionate double cycle** | |  |
| K01847 | MUT | methylmalonyl-CoA mutase [EC:5.4.99.2] |
| K01961 | accC | acetyl-CoA carboxylase, biotin carboxylase subunit [EC:6.4.1.2 6.3.4.14] |
| K01962 | accA | acetyl-CoA carboxylase carboxyl transferase subunit alpha [EC:6.4.1.2 2.1.3.15] |
| K01963 | accD | acetyl-CoA carboxylase carboxyl transferase subunit beta [EC:6.4.1.2 2.1.3.15] |
| K02160 | accB, bccP | acetyl-CoA carboxylase biotin carboxyl carrier protein |
| K15052 | K15052 | propionyl-CoA carboxylase [EC:6.4.1.3 2.1.3.15] |
| **Hydroxypropionic acid-hydroxybutyrate cycle** | |  |
| K01848 | E5.4.99.2A, mcmA1 | methylmalonyl-CoA mutase, N-terminal domain [EC:5.4.99.2] |
| K01849 | E5.4.99.2B, mcmA2 | methylmalonyl-CoA mutase, C-terminal domain [EC:5.4.99.2] |
| **Dicarboxylic acid-hydroxybutyrate cycle** | |  |
| K00626 | ACAT, atoB | acetyl-CoA C-acetyltransferase [EC:2.3.1.9] |
| K14534 | abfD | 4-hydroxybutyryl-CoA dehydratase / vinylacetyl-CoA-Delta-isomerase [EC:4.2.1.120 5.3.3.3] |
| **Reduction of acetyl-CoA pathway** | |  |
| K00198 | cooS, acsA | anaerobic carbon-monoxide dehydrogenase catalytic subunit [EC:1.2.7.4] |
| K00297 | metF, MTHFR | methylenetetrahydrofolate reductase (NADH) [EC:1.5.1.54] |
| K01491 | folD | methylenetetrahydrofolate dehydrogenase (NADP+) / methenyltetrahydrofolate cyclohydrolase [EC:1.5.1.5 3.5.4.9] |
| K01938 | fhs | formate--tetrahydrofolate ligase [EC:6.3.4.3] |
| K05299 | fdhA | formate dehydrogenase (NADP+) alpha subunit [EC:1.17.1.10] |
| K14138 | acsB | acetyl-CoA synthase [EC:2.3.1.169] |
| K15022 | fdhB | formate dehydrogenase (NADP+) beta subunit [EC:1.17.1.10] |
| K15023 | acsE | 5-methyltetrahydrofolate corrinoid/iron sulfur protein methyltransferase [EC:2.1.1.258] |
| **Nitrogen fixation** |  |  |
| K02588 | nifH | Nitrogenase iron protein NifH |
| K02586 | nifD | Nitrogenase molybdenum-iron protein alpha chain [EC:1.18.6.1] |
| K02591 | nifK | Nitrogenase molybdenum-iron protein beta chain [EC:1.18.6.1] |
| K00531 | anfG | Nitrogenase delta subunit [EC:1.18.6.1] |
| **Denitrification** |  |  |
| K04561 | norB | Nitric oxide reductase subunit B [EC:1.7.2.5] |
| K02567 | napA | Nitrate reductase (cytochrome) [EC:1.9.6.1] |
| K00371 | narH, narY, nxrB | Nitrate reductase / Nitrite oxidoreductase, beta subunit [EC:1.7.5.1 1.7.99.-] |
| K00370 | narG, narZ, nxrA | Nitrate reductase / Nitrite oxidoreductase, alpha subunit [EC:1.7.5.1 1.7.99.-] |
| K00368 | nirK | Nitrite reductase (NO-forming) [EC:1.7.2.1] |
| K00374 | narI, narV | Nitrate reductase gamma subunit [EC:1.7.5.1 1.7.99.-] |
| K00376 | nosZ | Nitrous-oxide reductase [EC:1.7.2.4] |
| **Assimilatory nitrate reduction(ANRA)** | |  |
| K00372 | nasA | Assimilatory nitrate reductase catalytic subunit [EC:1.7.99.-] |
| K00360 | nasB | Assimilatory nitrate reductase electron transfer subunit [EC:1.7.99.-] |
| K00366 | nirA | Ferredoxin-nitrite reductase [EC:1.7.7.1] |
| K00367 | narB | Ferredoxin-nitrate reductase [EC:1.7.7.2] |
| **Dissimilatory nitrate reduction(DNRA)** | |  |
| K15876 | nrfH | Cytochrome c nitrite reductase small subunit |
| K03385 | nrfA | Nitrite reductase (cytochrome c-552) [EC:1.7.2.2] |
| K00362 | nirB | Nitrite reductase (NADH) large subunit [EC:1.7.1.15] |
| **Nitrate assimilation** |  |  |
| K02575 | NRT, narK, nrtP, nasA | MFS transporter, NNP family, Nitrate/Nitrite transporter |
| K15576 | nrtA, nasF, cynA | Nitrate/Nitrite transport system substrate-binding protein |
| K15577 | nrtB, nasE, cynB | Nitrate/Nitrite transport system permease protein |
| K15578 | nrtC, nasD | Nitrate/Nitrite transport system ATP-binding protein [EC:7.3.2.4] |

**Table S2.** Degradation of the chemical properties of straw.

| Sample | Total K | Total N | Total P | Organic matter |
| --- | --- | --- | --- | --- |
|  | (g/kg) | (g/kg) | (g/kg) | (g/kg) |
| TU1 | 2.98±0.18abc | 6.28±0.17ab | 8.61±1.09ab | 640.70±65.78bc |
| TD1 | 3.30±0.17a | 5.97±0.99ab | 3.99±1.08c | 598.79±14.42c |
| TU2 | 2.28±0.17bc | 4.92±0.44ab | 6.10±0.69abc | 694.73±48.43ab |
| TD2 | 2.55±0.15abc | 4.40±1.04b | 5.12±0.43abc | 762.00±59.00ab |
| TU3 | 3.05±0.26ab | 6.10±0.52ab | 6.24±1.71abc | 617.54±44.48bc |
| TD3 | 3.30±0.61a | 7.05±1.07a | 7.87±0.61ab | 646.21±5.835bc |
| TU4 | 2.00±0.08c | 5.97±1.06ab | 7.95±0.94ab | 540.35±15.55bc |
| TD4 | 2.77±0.29abc | 4.58±0.30b | 8.77±0.33a | 582.25±24.38c |
| TU5 | 2.56±0.30abc | 7.18±0.56a | 5.35±2.31abc | 570.12±17.22bc |
| TD5 | 2.89±0.36abc | 5.54±0.16ab | 4.80±0.48bc | 542.55±15.28bc |

**Table S3.** Effect of nitrogen application and depth of return and its interaction on the process of degradation of carbon and nitrogen.
